# “There Are Too Many, but Never Enough": Qualitative Case Study Investigating Routine Coding of Clinical Information in Depression

**DOI:** 10.1371/journal.pone.0043831

**Published:** 2012-08-24

**Authors:** Kathrin Cresswell, Zoe Morrison, Aziz Sheikh, Dipak Kalra

**Affiliations:** 1 eHealth Research Group, Centre for Population Health Sciences, The University of Edinburgh, Edinburgh, United Kingdom; 2 Centre for Health Informatics and Multi-professional Education, University College London, London, United Kingdom; University of Pennsylvania, United States of America

## Abstract

**Background:**

We sought to understand how clinical information relating to the management of depression is routinely coded in different clinical settings and the perspectives of and implications for different stakeholders with a view to understanding how these may be aligned.

**Materials and Methods:**

Qualitative investigation exploring the views of a purposefully selected range of healthcare professionals, managers, and clinical coders spanning primary and secondary care.

**Results:**

Our dataset comprised 28 semi-structured interviews, a focus group, documents relating to clinical coding standards and participant observation of clinical coding activities. We identified a range of approaches to coding clinical information including templates and order entry systems. The challenges inherent in clearly establishing a diagnosis, identifying appropriate clinical codes and possible implications of diagnoses for patients were particularly prominent in primary care. Although a range of managerial and research benefits were identified, there were no direct benefits from coded clinical data for patients or professionals. Secondary care staff emphasized the role of clinical coders in ensuring data quality, which was at odds with the policy drive to increase real-time clinical coding.

**Conclusions:**

There was overall no evidence of clear-cut direct patient care benefits to inform immediate care decisions, even in primary care where data on patients with depression were more extensively coded. A number of important secondary uses were recognized by healthcare staff, but the coding of clinical data to serve these ends was often poorly aligned with clinical practice and patient-centered considerations. The current international drive to encourage clinical coding by healthcare professionals during the clinical encounter may need to be critically examined.

## Introduction

Information technology is increasingly used to facilitate the safety and efficiency of healthcare [1]. Systems rely upon real-time structured electronic data capture, often using coding (the activity of assigning a code to information in order to classify it), to process information in relation to, for example, performance measures, resource allocation, medical research, and billing [2]–[4]. In doing so, clinical statements (these may include medical diagnoses) within a classification system are assigned numerical values or clinical codes. There is therefore currently an increasing drive to achieve clinical coding by healthcare professionals at the point of care [5]. However, structured data entry systems also need to satisfy the clinical needs of users (often referred to as ‘clinical utility’) [6]–[9] and facilitate information exchange between different care settings in increasingly fragmented healthcare systems [4], [7], [10], [11].

Although in many countries coding of clinical information is used primarily for billing purposes (e.g. Canada and the United States of America (USA)), this is less common in the United Kingdom (UK) which has a publicly-funded national health service. Here, the coding of clinical information is focused on demographic details, diagnoses, reasons for encounters, numeric results, prescriptions, assessment scales and chronic disease monitoring datasets. The primary aims are to support care management, clinical decision making, adherence to guidelines, contributions to reimbursement, and less commonly, research. UK secondary care depression services use the ICD-10 (International Classification of Diseases 10th Revision) classification system for coding diagnoses, whilst GPs use Read Codes (a clinical coding system purchased by the UK government and used in primary care). Across countries, clinical coding is often carried out at the end of the consultation, after the healthcare professional has collected and recorded all relevant historical and examination data and there is an increasing drive towards adopting national terminologies that are drawn on across electronic systems facilitating data exchange between settings [12]. This poses significant new challenges for coding of clinical information when compared to paper-based record systems.

Mental health offers important insights into the challenges associated with electronic clinical coding and with classification systems: complex information is predominantly recorded as free-text narrative and diagnoses tend to be formulated over lengthy periods of time, which makes clinical coding of diagnoses extremely challenging. However, existing mental disorders classification systems may lack clinical utility [6], [7]. They are often perceived to fail to accommodate existing clinical practices as well as communication needs between care settings, resulting in a lack of immediate patient care benefits [6], [7]. This shortcoming is also recognized by the World Health Organization (WHO), which advocates that *“a mental health information system is a system for action: it should exist not simply for the purpose of gathering data, but also for enabling well-informed decision-making"* [4].

So why do coding and classification systems for mental disorders frequently fall short of clinical utility? Although there is a dearth of empirical work in this area, many have argued that existing systems do not appropriately reflect the reality and specific characteristics of mental healthcare [6], [13], [14]. They are often based on more physical diagnoses and are not well suited to accounting for multi-disciplinary, multi-agency care pathways [8], [13]. Concerns have also been expressed that diagnostic systems may mask the complexity of disorders and may lead clinicians to focus on symptoms present in a classification system, whilst potentially neglecting other significant factors such as contextual dynamics (e.g. social circumstances) [14]–[16].

Whilst clinical utility is clearly essential, it is also very important that managerial needs are met. We sought to explore how a balance in this respect may be achieved and if and how mental health information systems contribute to clinical decision-making. In doing so, we explored how clinical information relating to depression is structured and/or coded in primary and secondary care settings and the viewpoints of, and implications for, different stakeholders.

## Materials and Methods

As mental health is a large area, we focused on the area of depression as one of the most common mental disorders with numbers steadily increasing worldwide [16], [17]. Internationally, depression is usually diagnosed and treated in primary care [18]. Clinical codes across care settings are most commonly mapped onto the ICD-10 (developed by the WHO) and the Diagnostic and Statistical Manual of Mental Disorders, Fourth Edition published by the American Psychiatric Association [19]–[21].

### Design

We conceptualized the disorder depression as a case in order to focus our data collection activities. This case study design allowed us to explore the phenomenon of interest (i.e. routine coding of clinical information in depression) within context, drawing on multiple sources of evidence.[22]–[26] We collected qualitative data from a range of stakeholders and geographical locations, spanning primary and secondary care settings in the UK over a period of four months. Exploring a range of NHS information structures within the UK (i.e. primary and secondary care settings) and obtaining data from a variety of qualitative data sources, allowed us to gain an insight into context-specific and cross-cutting issues relating to clinical coding in depression within one political setting.

Our data collection and analysis strategy was theoretically informed drawing on sociotechnical principles [25], [26]. In essence, this approach emphasizes the interrelated nature of social and technical dimensions and we therefore sampled individual participants on the basis of their relationship with coding of clinical computerized information relating to depression, exploring how individual behaviors shaped technical outputs and vice versa.

### Ethical and Site-specific Approvals

We obtained ethical approval from the Brighton West Ethics Committee (Reference: 10/H1111/25). Site-specific approvals, advanced disclosures and honorary contracts were obtained for the lead researcher (KC). Written informed consent was obtained from all participants and data obtained were anonymised.

### Recruitment of Participants

We initially purposefully sampled a range of academic General Practitioners (GPs) with an interest in clinical coding and/or depression through personal contacts [27]. Interviews with academic GPs helped to provide an insight into secondary uses of data for research purposes and a preliminary overview of clinical coding practice as well as tensions in the primary care setting. Participants were asked to recommend other potential informants, which helped us to snowball sample additional primary care stakeholders including those with no particular interest in clinical coding as well as clinical coders.

We also recruited secondary care staff at a mental health Trust (our main secondary care research site) identified through the UK Clinical Research Network [28], purposefully sampling diverse stakeholders with an insight into depression and/or clinical coding practices, including healthcare professionals (i.e. nurses, doctors, allied health professionals), managers and clinical coders [27]. This allowed exploring the dynamics of everyday clinical depression coding from a variety of perspectives. Participant characteristics and their involvement in data collection activities are summarized in Table 1.

**Table 1:** Participant characteristics and their involvement in data collection activities.

Participant number	Profession	Type of data collected	Setting
1	Academic GP	Interview	Primary care
2	Academic GP	Interview	Primary care
3	Academic GP	Interview	Primary care
4	Clinical Coding Tutor from a NationalInformation Services Division	Interview	Cross-cutting
5	Academic GP	Interview	Primary care
6	Academic GP	Interview	Primary care
7	Academic GP	Interview	Primary care
8	Mental Health Welfare Commission representative	Interview	Cross-cutting
9	Academic GP	Interview	Primary care
10	Consultant Psychiatrist	Interview	Secondary care
11	GP	Interview	Primary care
12	Data Entry Clerk (primary care)	Interview	Primary care
13	GP	Interview	Primary care
14	GP	Interview	Primary care
15	Academic GP	Observation	Primary care
16	Quality and Outcomes Framework Manager(primary care)	Interview	Primary care
17	Consultant Psychiatrist	Interview	Secondary care
18	Centre Manager (nursing background)	Interview	Secondary care
19	Research Nurse	Interview	Secondary care
20	Ward Manager (nursing background)	Interview	Secondary care
21	Information Service Manager	Interview	Secondary care
22	Clinical Coding Manager and two Clinical Coders	Focus Group	Secondary care
23	Consultant Geriatrician	Interview	Secondary care
24	Cognitive Behavioral Therapist	Interview	Secondary care
25	Physiotherapist	Interview	Secondary care
26	Occupational Therapist	Interview	Secondary care
27	Centre Manager (nursing background)	Interview	Secondary care
28	Consultant Psychiatrist	Interview	Secondary care
29	Nurse Practitioner	Interview	Secondary care
30	Consultant Psychiatrist	Interview	Secondary care

### Data Generation and Handling

Our work was informed by a review of the existing literature and involved a combination of 28 semi-structured face-to-face and telephone interviews (our main data source), a focus group with clinical coders, a two-hour period of participant observation of selected clinical coding activities in primary care, and collection of documents to aid understanding of context (e.g. information on clinical coding standards). We gathered data from a variety of data sources to facilitate the credibility of our findings (triangulation). Our overall dataset is summarized in Table 2.

**Table 2:** Summary of data collected.

Primary care	Secondary care	Cross-cutting
Interviews with seven academic GPs,three non-academic GPs, a Qualityand Outcomes Framework manager,a primary care data entry clerk	Interviews with four consultant psychiatrists, twocenter managers with nursing backgrounds, a researchnurse, a ward manager with nursing background,a nurse practitioner, an information service manager,a consultant geriatrician, a cognitive behavioraltherapist, a physiotherapist, an occupational therapist	A representative from the mental health welfare commission, a clinical coding tutor from a national information services division
An observation of clinical coding activitylasting two hours	A focus group with three clinical coders	30 field notes
		Seven documents relating to information on clinical codes and/or structuring standards

Key issues explored included attitudes towards clinical coding in relation to depression; perceived benefits, barriers and facilitators; and recommendations for improvement (Table 3).

**Table 3:** Sample interview guide.

Main structure	Specific topics and issues
Confidentiality, aims, thanks	Theorized and actual benefits and risks, drivers, incentives, barriers and how to address these
Any questions?	
**About yourself**	Role, do you capture and store health information yourself and, if yes, what and how? (setting, profession, clinical coding system, electronic system)
Main **drivers for structuring and/or coding clinical information**in depression	In what **instances is structured and/or coded clinical information really helpful**? What **impact** does the use of structures and/or clinical codes have **on clinical care and outcomes, or on patient experience and engagement**?
Do the **structures and/or clinical codes cover what you feel needs** **to be recorded** – any **areas for improvement**?	In terms of **completeness and accuracy** and in terms of **enabling good use of the information** Any potential **uses of the information that are under-exploited**? If yes, why?
**Overall**	
How well do the available clinical systems support structuring and/orencoding the clinical information?	
Any **other barriers** to collecting good quality information?	
Any **drivers or incentives** that would improve the quality or usesmade of this information?	
Any **international developments** in relation to structuring and/orcoding clinical information in depression they are aware of?	Any examples of innovation/centers of excellence?
Aware of any other areas e.g. prisons, learning disability, homelessshelters and clinical coding there?	
**Concluding remarks**	
Anything else?	
Thanks, any questions?	
Anyone they can recommend for interview?	
Any relevant literature?	

Topic guides were tailored to the roles of individual participants and refined throughout the research, with emerging issues being recorded as field notes and used to inform subsequent rounds of data collection. Interviews were informal and centered on issues that interviewees perceived to be important.

A focus group with clinical coders working in secondary care and participant observation of clinical coding in primary care were undertaken to complement interviews and provide greater understanding of the context in which coding decisions are made [29]. The observation involved the researcher practicing clinical coding on dummy patients (i.e. coding diagnoses and symptoms on fictional patients that were used for training system users locally) whilst discussing activities with the relevant healthcare professional. This exercise helped to provide insights into the experiential aspects of clinical coding activity and facilitated the credibility of our findings. Data collection continued until no new themes emerged. Transcribed interview and focus group data, as well as observation and field notes were uploaded into NVivo8 software [30].

### Analysis

Data collection and analysis took place concurrently to allow emerging issues to be fed back into future data collection. A coding framework was developed and refined based on the topic guide and literature review to include the following themes: background and context (relating to systems, interviewees and settings); existing practices; definitions and diagnoses; facilitators and barriers to structuring and/or coding of clinical information in depression; perceived benefits; and recommendations for improvement. In addition to this deductive approach, inductive approaches were employed to allow new themes to emerge from the data [31]. The lead researcher (KC, a psychologist with experience in qualitative health services research, particularly in relation to the use of IT in healthcare) coded the data, combining different data sources within the coding framework in order to look for patterns of convergence, maintaining a field journal throughout the process in order to capture key analytical processes. Existing and emerging findings were discussed in designated analysis workshops with the extended research team exploring inconsistencies and unexpected findings seeking novel and potentially unexplored evidence [32]. Analytic themes were extracted based on frequency of occurrence and relevance.

## Results

Three main themes emerged from the analysis (Table 4). These themes are examined in detail below, illustrated with quotes from the data.

**Table 4:** Summary of main themes and sub-themes.

Varying contexts and practices surrounding the coding of clinical information in depression.
– Wide variations in care pathways resulting in the need for free-text entries in addition to coded clinical information.
– Differences in type and methods of coding clinical information between primary and secondary care settings.
– Differences in information technology systems and implications for associated clinical coding practices surrounding degrees of autonomy in selecting and tailoring clinical codes.
– Lack of unified clinical coding system between primary and secondary care settings.
Lack of direct patient care benefits and a number of risks surrounding the coding clinical data in depression.
– Drawing on coded clinical information for management and research purposes.
– Lack of contribution of coded clinical information to clinical decision-making and direct patient care.
– Lack of understanding amongst healthcare professionals surrounding the value and use of coded clinical data.
– Retrospective coding of clinical information.
– Loss of contextual information and the value of free text.
– Diagnostic rigor and uncertainty in complex mental health conditions.
– Tensions surrounding the number and meaningful arrangement of clinical codes.
Strategies employed to align clinical value with managerial demands.
– Motivations and incentives to code clinical information.
– Tailoring of systems versus standardization and the role of templates.
– The role of clinical coders in secondary care settings – consistency of interpretation.

### Varying Contexts and Practices Surrounding the Coding of Clinical Information in Depression

Most participants stated that contexts and conditions in depression were distinct from other *“more biomedical"* areas of care: disorders were felt to be difficult to define, care occurred over periods of time with often changing pathways, and wide variations in responsiveness to treatment. Consequently, there were often no prescribed care pathways once a diagnosis of a mental disorder had been made.

Although certain information would be coded (e.g. symptoms and diagnoses), this was almost always accompanied by free text (e.g. investigation findings and treatment plans) to reflect the subtleties of a patient’s situation and the thoughts and feelings of the practitioner.

“…actually often a mental health consultation will just exist in history, and in that history chunk will be…feeling more depressed this time, what’s been going on…and even my thoughts and formulation may all just sit in that box [of free text] because actually it comes out of my head in one chunk that is…three or four lines of text."

(Interview 1, Academic GP).

GPs coded clinical information relating to symptoms, diagnoses, history, and investigations. In the UK, depression is included in a centrally-led payment incentive scheme, the Quality and Outcomes Framework [33], which requires GP practices to submit activity levels in relation to certain conditions to commissioners in order to get paid.

Secondary care clinical coding included:

•Interventions/activities (e.g. assessment of mental state, cognitive testing) – nurses and allied health professions;
•Admission diagnoses/review and discharge diagnoses - doctors for inpatients only;
•Clustering adult mental health users to diagnostic pathways – nurses and allied health professions.

We found the use of computer systems and associated clinical coding practices to be well established in UK primary, but regarded as a relatively recent development in secondary care. Perhaps as a result of this, GPs did some clinical coding during the clinical encounter, whilst in our secondary care site doctors’ diagnoses were coded by a designated clinical coding team (although some clinical intervention and cluster coding was done by other healthcare professionals). The fact that secondary care depression services and GPs used different coding and classification systems, was attributed to differing patient needs. Whilst the majority of depression is diagnosed and treated in primary care, secondary care tends to manage the more severe spectrum of depressive disorders and may therefore need more granular diagnostic classification:

“I mean psychiatrists have always had lots of codes…the fine grained detail in my opinion of diagnosis at that level is more relevant than it is in primary care."

(Interview 1, Academic GP).

Differences in systems and associated clinical coding practices were apparent in both primary and secondary care settings. Such systems variations made it more difficult to obtain comparable data sets for research purposes:

“…that’s another problem that we’re encountering with our research cos we’re getting information from…sort of twenty odd GP surgeries in South London and there’s striking differences between [name of a primary care computer system] which is by far and away the best and these other systems which are chaotic in terms of how they organize…information."

(Interview 7, Academic GP).

In some systems, users could ignore requests to code clinical information and focus only on free text. This was commonly the case in primary care, facilitating greater user autonomy. Our main secondary care research site had implemented a “home-grown" computer system which mandated some clinical coding by nurses and allied health professions preventing the user progressing to a different screen unless a clinical code had been picked. The “home-grown" nature was in many ways perceived to be an advantage, as it was relatively easy to tailor associated clinical codes.

“…we can develop the system to capture what we need very quickly, it’s quite responsive and that’s kind of put us streets ahead of the other Trusts in that respect."

(Interview 19, Research Nurse, Secondary Care).

At the time of data collection, there was no unified clinical coding system between primary and secondary care and no software to map between clinical codes. Upon transfer between care settings, patient information had to be re-coded, which was viewed as a duplication of effort.

“They [GPs] all use different [clinical codes] and their computer system doesn’t link up with ours so none of the codes match across primary and secondary…"

(Interview 24, Cognitive Behavioral Therapist, Secondary Care).

### Lack of Direct Patient Care Benefits and a Number of Risks Surrounding the Coding Clinical Data in Depression

The perceived benefits of coding clinical information were mainly noted as the opportunity to readily access information for management and research purposes. Coded clinical information was also suggested as being useful for clinical audits (performed as an internal peer review activity), not only for organizations, but also for individual teams as it allowed an insight into activity levels, helped to monitor patient populations, and to prioritize the allocation of resources.

“It’s about teams understanding their own activities…admission rates, discharge rates, average patient stay, average age of patients…which from a team point of view is very helpful for us to understand what’s going on in any given month really…"

(Interview 20, Ward Manager with nursing background, Secondary Care).

Despite these indirect benefits to patients, most clinically practicing interviewees stated that clinical coding was designed to achieve organizational targets and offered very little value for direct patient care.

“Clinically it feels like you’re doing it really for the organization rather than for any benefits for the patients… it’s just time out that’s not helpful, it feels like you’re feeding information back…for commissioning purposes so that you get paid basically rather than it being a clinically useful tool."

(Interview 24, Cognitive Behavioral Therapist, Secondary Care).

Many clinical users therefore felt detached from the development of clinical codes, stating that coded clinical data input was a time-consuming administrative activity, which often detracted from the focus of delivering patient care. This was exacerbated by many clinical users, particularly those in secondary care, being unsure as to why they needed to record coded clinical information and what data were used for.

On a practical level, clinical users struggled to juggle the demands of data input with delivering adequate patient care for the complex demands of depressive patients in the limited time available. This was particularly pronounced in primary care, where consultations were shorter than in secondary care. Clinical coding was often viewed as a bureaucratic exercise with no particular purpose in itself and a lack of meaningful representation of clinical activity.

“… you try and talk to your patient and then quickly enter everything on to the template. But you have ten minutes. Before somebody comes in and interrupts you, will you just sign this prescription, then the phone rings and somebody says oh doctor there’s somebody in treatment room, I think they’ve got cellulitis, will you just run across and see if they need antibiotics. So you do that, so you’ve then got six minutes. For a psychiatric patient, to assess them and make all the notes. It’s not actually possible."

(Interview 13, GP).

Consequently, notes were often taken on paper and coded after the clinical encounter. This was felt to be necessary in depressed patients as there was often a significant amount of counseling involved in encounters and coding clinical information was viewed as disrupting communication flows.

“We just remember it and then at the end of the day put it in the computer who you saw and what you did, perhaps do a scribbly note to yourself of who you saw."

(Interview 26, Occupational Therapist, Secondary Care).

“… when we are seeing patients we have to talk the patient we can’t be actually sitting in front of the computer and typing…, you need to communicate with the patient you can’t just do the typing while you are taking to patients."

(Interview 30, Consultant Psychiatrist, Secondary Care).

Other underlying reasons for negative attitudes towards clinical coding amongst healthcare professionals were related to the perceived particularities of mental health disorders. For example, many interviewees raised the difficulty of fitting complex mental health conditions into coded clinical categories as they felt that context was often lost. Information reflected in free text was therefore valued, whilst clinical coding was perceived to facilitate labeling and force-fitting patients into boxes.

“Sometimes people just don’t fit the coding…and you’re actually trying to fit them into the ICD-10 rather than the ICD-10 being created to match the general public really… sometimes you are putting a square peg in a round hole."

(Interview 29, Nurse Practitioner, Secondary Care).

Clinicians found it hard to choose an appropriate clinical coding category, often picking the closest match as opposed to what they felt to be an accurate description.

As a result of perceived issues surrounding the rigidity of applying labels to complex mental health conditions, clinicians (particularly in primary care) were often hesitant to apply diagnostic codes due to the uncertainty surrounding the persistence of symptoms and the potentially contestable nature of diagnoses.

This reluctance was further exacerbated by the potential ramifications of diagnoses beyond the clinical care setting, such as social stigma, inclusion in the mental health register, implications for insurance reports and occupational health screening.

Diagnostic coding was further complicated by perceived issues surrounding the definition of depression, due to its multi-dimensional nature and the absence of biomarkers. For example, GPs highlighted the need to be cautious in applying diagnostic labels as some individuals presenting with depressive symptoms may be merely experiencing a normal reaction to adverse life events.

“… you have to be careful with labeling people. And yes it might be you know is it a depressive episode because their serotonin levels are low? …is it because actually their partner’s just lost their job and finances are tight and actually it’s stress and worry about that which isn’t depression."

(Interview 11, GP).

Participants across care settings further expressed a tension relating to the number of clinical codes IT systems offered. On one hand, existing clinical code lists were felt to be too detailed resulting in a large number of irrelevant items, whilst, on the other hand, available clinical codes often did not accurately reflect diagnoses or interventions.

“…there’s anxiety with depression, there’s recurrent depression, there’s depressed mood, there’s loads of them, ok? So you have to select a suitable code and…they’re never exactly right …there’s either too many of them so you don’t know which one to choose or there’s not enough because the one circumstance that you’ve got sitting in front of you isn’t the one that’s got a code."

(Interview 5, Academic GP).

### Strategies Employed to Align Clinical Value with Managerial Demands

Despite these difficulties and the overall perceived lack of direct patient care benefits resulting from clinical coding, when prompted, some participants valued the ability to obtain an overview of clinical information on the summary screen, the ability for different healthcare professionals to share patient information, the ability to use of coded clinical information for clinical decision support systems, and the potential of facilitating medication reviews (e.g. allowing to link problems to medications). Despite these perceived benefits, across care settings, the main incentives for clinical coding information were financial.

“I don’t see any value in encoding any specific bits of that other than a particular depression score which I get paid for recording and why else would I do it?"

(Interview 1, Academic GP).

“I think people will probably be more likely to code better. If people realize that they’re not going to get paid unless they’ve actually done things for the patient they’re more likely to put correct stuff in…"

(Interview 25, Physiotherapist, Secondary Care).

In addition, across care settings the potential of clinical coding to facilitate adherence to quality indicators was valued in the context of using templates, which were perceived to be able to guide clinical decision making by ensuring that appropriate aspects were considered. Templates, devised by individual organizations relating to various conditions and/or for different practitioners, were also perceived to address the tension between tailoring of systems to suit individual practices and the need for some degree of standardization to ensure the usefulness of coded clinical data for management and research.

“I would say that [GPs] are not interested in inputting codes…I think I’ve got more chance of them using the templates, clicking the hot spots and it automatically coding it than actually inputting the coding themselves."

(Interview 16, Quality and Outcome Framework Manager).

Moreover, and mainly in secondary care settings, clinical coders were employed to take over the responsibility for the coding of clinical information. Most clinicians stated that they would prefer clinical coders to input information for them as they were busy with providing patient care. This was seen as an acceptable compromise on quality (as their clinical coding may not be as precise as that of clinicians) and utility (as the benefit from real-time clinical coding may be lost). Others stated that clinical coding by clinicians at the point of care provided a more timely and up-to-date patient record than retrospective clinical coding by clinical coders. Increasing clinical coding by clinicians at the point of care was felt to have the potential of addressing inaccurate retrospective clinical coding by non-clinical staff.

Some participants, notably those that worked with coded clinical data outputs, also argued that clinical coders were particularly valuable in ensuring consistency. Clinical coders were viewed as experts in the field of allocating clinical codes, which was not the expertise of healthcare professionals.

“I’m sure there’s a whole host of problems, you know, without us directly inputting the information but I do know when we were inputting things into the system we were getting it wrong which meant that it was causing more problems hence it being taken back off us for the clinical information team to do."

(Interview 20, Ward Manager with nursing background, Secondary Care).

Similarly, clinical coders were often highly valued by clinicians as an expert resource and safety-net:

“…generally it [referring to clinical coders checking diagnoses with clinicians] also acts for the clinicians to avoid that mistake happening again so it becomes educational and then reduces further errors."

(Interview 30, Consultant Psychiatrist, Secondary Care).

## Discussion

### Summary of Main Findings

This work has revealed the range of approaches to coding clinical data on patients with depression and how these are shaped by varying contexts, information systems and practices. We have outlined how, despite the value of coding clinical information relating to depression for management and research purposes, there is a perceived lack of direct patient care benefits and a number of risks associated with clinical coding practices in this area. In exploring strategies employed to align clinical value with managerial demands, we have discussed the underlying clinical motivations to code clinical information relating to depression, as well as the potential value of employing templates and clinical coders.

### Strengths and Limitations

As far as we are aware, this work is the first in-depth empirical study of the approaches to coding clinical information in depression and our findings are we believe likely to be transferable to other mental health conditions and international settings. The likely transferability of this work is further reinforced by the existing empirical literature surrounding clinical coding in other conditions, which has pointed to related concerns and issues [34]–[38]. Drawing and building on sociotechnical approaches to technology implementation employed in previous work [25], [26], has allowed a theoretically informed basis for sampling and analysis. For the period of time we spent in the field we have, we believe, reached saturation as we continued data collection until no new themes emerged.

Nevertheless, there are also some limitations including a lack of insight into system developers’ perspectives due to commercial sensitivities and our initial focus on interviewing academic GPs, which was based on pragmatic considerations, may have biased our focus towards issues that are not immediately visible to non-academic practicing clinicians (e.g. they might have attached greater value to the benefits associated with the secondary uses of data for research purposes). Our exploratory focus in an area that lacked empirical insights meant that interviews were appropriate in the context of our study. However, exploring how best to integrate different systems within complex individual user workflows and derive maximum benefits for all concerned, warrants the need for some more targeted in-depth observational work in individual settings.

### Considering our Findings in the Light of the Existing Literature

We have investigated the often neglected perspectives of clinical coders and considered potential trade-offs between clinical coding by healthcare professionals during the clinical encounter and retrospectively by clinical coders. Although there is an international drive to increase clinical coding at the point of care to improve data quality [5], our results suggest that this may in practice reduce data quality by increasing inconsistencies in clinical coding. The intuitive assumption that clinical coding at the point of care is preferable should therefore be questioned.

Our results confirm the potential value of coded clinical data for managerial and research purposes, but direct patient care benefits are currently lacking as existing systems (e.g. ICD) are often designed primarily for reimbursement [3], [39]. This is despite most clinical coding efforts being invested by healthcare professionals, associated risks such as a perceived reduction in the quality of patient contact due to increased administrative activity, and concerns regarding the implications of coding patient diagnoses of often stigmatizing mental health conditions. Other systems, using clinical vocabularies (e.g. Systematized Nomenclature of Medicine-Clinical Terms, SNOMED-CT) to document,[19], [37] can be more valuable for clinical users as the clinical codes are often captured by the system as a largely background activity, later drawn upon by clinical coding professionals for other purposes. The question is therefore, whether and how clinical coding activity can be optimized by giving different sets of professionals the right tool for their respective purposes.

Different mental disorders may have diverse and overlapping biological and experiential causes [8], [16]. Despite an increasing recognition that classification and clinical coding systems need to take this complexity into account, existing systems are often viewed as inadequate resulting in inaccurate and/or unrepresentative clinical coding [3], [7], [8], [15]–[17], [40]–[42]. These tensions illustrate the need to balance classification systems without masking the unique complexity of mood disorders. These discussions are particularly relevant in the primary care setting as diagnostic decisions more contestable at the less severe end of the depressive spectrum.

Tailoring systems to individual needs, be it countries, care settings, organizations, or individual users, has frequently been proposed in the literature as a way to facilitate adoption [6], [8], [43]. This may entail giving users and/or organizations the authority to priorities categories in line with perceived need and increased involvement in the development of clinical codes [4], [43]. Our findings highlight the need to achieve a balance between tailoring of systems to promote clinical utility and a degree of standardization to allow meaningful analysis of data on a larger scale. One solution may be the combination of coded clinical data entry with free text, which has been found to improve categorization rates amongst users [44].

We have further illustrated the importance of financial incentives in promoting clinical coding activity across care settings [45], [46]. In the literature, there is some evidence that linking healthcare professional performance to payment can improve the quality of care [47]–[49], but there remains a need to explore alternative incentives, such as feedback on user performance – otherwise conditions not included in payment schemes may suffer [47]–[49].

Tensions between the need to standardize systems whilst maintaining clinical utility were also illustrated by debates surrounding the number of categories in existing mental disorders coding and classification systems. Participants felt that subcategories were becoming increasingly specific as this was desirable for research and management purposes. However, this meant that clinical users were faced with rising numbers of terms, which they often found time-consuming to browse and difficult to remember. Consequently, and in line with our work, existing studies have found that many diagnostic categories in psychiatric settings are not used at all and there is a high incidence of unspecified diagnoses [6], [7], [50]. A potential solution, frequently mentioned in the literature and also by our participants, could be to reduce the number of diagnostic categories [7], [35], [38], [51], [52], which may be achieved by grouping these together around meaningful clusters [7], [14], [41]. However, the ultimate decision will not only depend on clinical utility, but will also need to be decided on the basis of, amongst other issues, public health utility, which generally requires more granularity [8], [41].

### Potential Ways Forward

Based on our work, we have a range of recommendations, which policy makers may wish to consider when deliberating strategic directions in relation to clinical coding of information in the UK mental health setting and beyond (Table 5).

**Table 5:** Summary of recommendations emerging from this case study.

**Strategic**
– Definitions relating to both clinical terms and related content to be based on an agreed set of criteria to ensure consistency in clinical coding practices.
– Agreed clinical coding standards between care settings to facilitate information exchange.
– Aligning different existing clinical coding and classification systems.
– Updating structures and/or clinical codes in pace with professional practices.
**Development of Systems**
– Devise systems that are meaningful in relation to their purposes (clinical activity, secondary uses).
– Arranging categories in more meaningful clusters (ideally based on hierarchies).
– Reducing the number of categories – particularly in primary care.
– Balancing structured and/or coded entry and free text based on severity of symptoms and settings.
– Devising systems that facilitate meaningful clinical coding of interpreted syndrome components.
– Devising clinical coding systems that capture relationships as well as clinical codes to derive most value out of systems (e.g. changes in diagnoses over time).
**Organizational**
– Clinical coding champions to ensure consistent approaches to clinical coding amongst teams.
– Promote the relationship between clinicians and clinical coders by placing them as close to clinical work as possible.
– Pay attention to professional differences in clinical coding practices.
– Training of healthcare professionals in clinical coding practices and increased involvement in development of clinical codes.
– Agreement on clinical coding practices within individual settings and/or health communities to ensure consistency.
– Explore and encourage innovative approaches to clinical coding e.g. voice recognition, portable hardware.

In terms of overall strategy, there is a need to agree centrally determined clinical coding standards and align systems between care settings to facilitate information exchange. This will require some degree of tailoring of systems to local needs to allow effective integration within work practices. National and international quality improvement initiatives have an important potential role to play to ensure clinical coding in line with evidence-based practice.

The option of free text information capture in mental health settings is likely to be of continuing importance, but there may be value in considering a balance of coded clinical data entry and free text based on severity and type of diagnoses. For instance, whilst mild depression may need an increased amount of free text as life circumstances are often important, severe depression may be more amenable to clinical coding as patients are most likely to benefit from standardized treatments. Systems which facilitate coding of symptoms initially, followed by coding of diagnoses, would help to address the evolving nature of mental health conditions.

Finally, organizations themselves may wish to consider drawing more explicitly on the expertise of local clinical coding teams, who can advise on how best to derive clinical value from systems. Throughout, recognition of professional differences in clinical coding practices is necessary as some professions and their associated nature of work may be more amenable to clinical coding than others.

### Conclusions

Our work has highlighted important aspects relating to coding clinical information in the mental health setting. In line with the existing literature, we found that this area differs in important ways from the more biomedical conditions with issues surrounding definitions, cut-off points and the evolving nature of mental disorders. We have also raised some questions relating principally to the implicitly assumed benefits of coding and classification systems. We found these to be lacking in the immediate clinical context and in relation to the drive to encourage clinical coding by healthcare professionals during the clinical encounter. Based on our findings, we have made a number of suggestions that may be considered to address existing issues and explored these from a range of strategic, technical, and organizational angles.

Overall, we feel that it is important to now take a step back and re-consider our own implicit assumptions about effectiveness and benefits, recognizing that these may be associated with significant trade-offs. This should involve deliberating why and for what purpose information is gathered on different levels including large-scale (e.g. epidemiological), organizational (e.g. management), and micro-environmental (e.g. the clinical encounter). The first step to achieving this, will involve formulating a challenge and then develop technical solutions to address this. Otherwise there is a danger that clinicians are asked to spend valuable clinical time on coding clinical information which may never be used.
