# Correction: “There Are Too Many, but Never Enough": Qualitative Case Study Investigating Routine Coding of Clinical Information in Depression

**DOI:** 10.1371/annotation/48c1ae80-d961-4c30-b966-243f010a0a1b

**Published:** 2012-10-26

**Authors:** Kathrin Cresswell, Zoe Morrison, Dipak Kalra, Aziz Sheikh

The authors are listed out of order. Please view the correct author order, affiliations, and citation here:

Kathrin Cresswell^1*^, Zoe Morrison^**1**^, Dipak Kalra^**2**^, Aziz Sheikh^**1**^

**1** eHealth Research Group, Centre for Population Health Sciences, The University of Edinburgh, Edinburgh, United Kingdom, **2** Centre for Health Informatics and Multi-professional Education, University College London, London, United Kingdom

Cresswell K, Morrison Z, Kalra D, Sheikh A (2012) “There Are Too Many, but Never Enough": Qualitative Case Study Investigating Routine Coding of Clinical Information in Depression. PLoS ONE 7(8): e43831. doi:10.1371/journal.pone.0043831

